# Cost-Effective Sampling Strategies for Wastewater Surveillance: A Large-Scale Longitudinal Study in Hong Kong and Shenzhen

**DOI:** 10.1021/acs.est.5c02652

**Published:** 2026-03-06

**Authors:** Xiawan Zheng, Yinghui Li, Yu Deng, Bincai Wei, Xiaoqing Xu, Chen Du, Guixian Luo, Miaomiao Luo, Xiuyuan Shi, Yuejing Peng, Shuxian Li, Jiahui Ding, Bingjie Xue, Yanping Mao, Qinghua Hu, Tong Zhang

**Affiliations:** † Environmental Microbiome Engineering and Biotechnology Laboratory, Center for Environmental Engineering Research, Department of Civil Engineering, 25809 The University of Hong Kong, Pokfulam, Hong Kong SAR 999077, China; ‡ 568734 Shenzhen Center for Disease Control and Prevention, Shenzhen 518073, China; § College of Chemistry and Environmental Engineering, Shenzhen University, Shenzhen 518071, China; ∥ School of Public Health, LKS Faculty of Medicine, The University of Hong Kong, Pokfulam, Hong Kong SAR 999077, China; ⊥ Department of Environmental Science and Engineering, Macau University of Science and Technology, Taipa, Macau SAR 999078, China; # School of Public Health and Emergency Management, 255310 Southern University of Science and Technology, Shenzhen 518055, China; ∇ Shenzhen Innovation and Research Institute, The University of Hong Kong, Shenzhen 518057, China; ○ School of Public Health, Southern Medical University, Guangzhou 510515, China

**Keywords:** SARS-CoV-2, wastewater surveillance, sampling strategy, sampling frequency, public health

## Abstract

During the COVID-19 pandemic, wastewater surveillance undoubtedly served as a useful public health surveillance tool and catalyzed the establishment of over 4600 sampling sites and 195 wastewater surveillance dashboards worldwide. However, in the postpandemic era, the continuous regular operation of these sites has become labor-intensive and costly. In this study, we established two downsampling strategies (i.e., Enumerative Method and Iterative Hierarchical Method) to explore the potential of reducing the number of sampling sites and sampling frequencies without significantly affecting the observed SARS-CoV-2 transmission trends. To evaluate the method’s effectiveness, we comprehensively applied them to two intensive large-scale wastewater surveillance data sets from two adjacent major cities, i.e., a 9-month monitoring across 12 sampling sites in Hong Kong and a 5-month monitoring across 38 sampling sites in Shenzhen. We found that consistent citywide SARS-CoV-2 transmission trends were captured in the two cities before and after halving the sampling sites. Additionally, we observed that multiple factors (i.e., flow rate, serving population size, number of sampling sites, geographical locations, etc.) influenced the site selection and trend representativeness, which should be comprehensively considered in the design of a sampling strategy. Moreover, sampling three times per week could reflect the virus transmission patterns during the Omicron outbreak. These findings highlight the necessity to optimize current sampling practices in most wastewater surveillance networks so that they can maximize the value of wastewater surveillance and achieve favorable cost-effectiveness for sustainable long-term monitoring.

## Graphical abstract

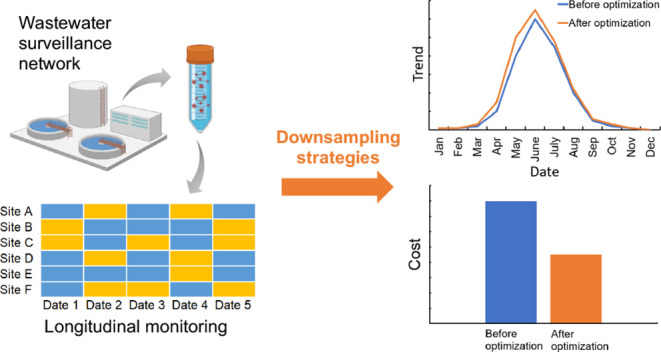

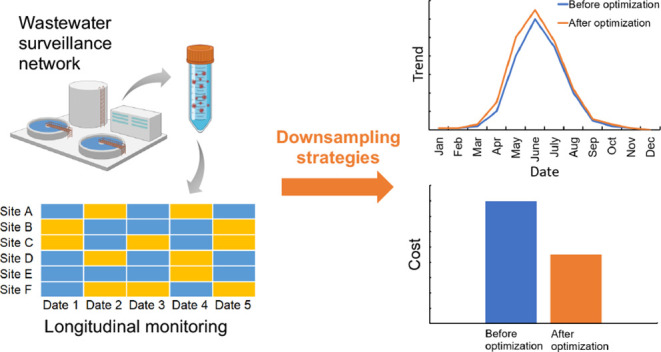

## 1 Introduction

Wastewater-based epidemiology (WBE), also known as wastewater surveillance (WWS), is a promising tool for conducting large-scale population testing to complement public health interventions in multiple aspects, such as monitoring the spread of infectious viruses (e.g., SARS-CoV-2, influenza virus, mpox virus, and diarrhea viruses),^1–3^ identifying the transmission of cryptic variants,^4,5^ and predicting epidemiological parameters for healthcare system preparedness.^6,7^ As of February 2026, the success of WBE applications has substantially increased the number of sampling sites to over 4600 across 72 countries worldwide.^8^ These sampling sites have undeniably provided crucial public health insights for controlling virus transmission during the COVID-19 pandemic, especially when clinical surveillance is inadequate, inaccessible, and costly. However, the long-term testing practices of these sampling sites impose a heavy burden in terms of costs, labor, and participation willingness in many regions and countries. Consequently, there is a growing concern regarding the necessity of operating all these sampling sites in the postpandemic era. The design of sampling strategies should strike a balance between surveillance purposes and limited resources, especially when it comes to a practical public health tool rather than academic research within laboratories. In this context, a pilot study aimed at optimizing the current sampling strategy can not only reduce the costs and labor required for long-term longitudinal wastewater surveillance but also offer new insights into the methodology of wastewater surveillance for monitoring other pathogens.

Sampling strategy is a fundamental component to initiate, establish, and implement WWS campaigns, which should be fit-for-purpose, cost-effective, and aligned with financial and organizational constraints.^9^ Previous studies have preliminarily evaluated the sampling frequency during the early stage of the pandemic,^10,11^ but there is still no consensus guidance regarding the selection of sampling sites. In addition, the sampling frequency needs to be optimized for different surveillance purposes, the emergence of new variants, the surveillance locations, etc. Therefore, more research studies are needed to address these research gaps. However, the evaluation of different sampling strategies relies on available long-term data sets and standardized methods to ensure the robustness of comparison results. To date, limited studies have been reported to address this bottleneck due to the lack of such data sets. As two adjacent major cities, Hong Kong and Shenzhen experienced similar pandemic trajectories and adopted similar methods to detect SARS-CoV-2 virus concentration in wastewater samples,^12,13^ which provides a great opportunity for us to explore the efficacy of different sampling strategies.

In the present study, we evaluated the impact of different sampling choices on sampling sites and sampling frequencies to understand how sampling choices affect outcomes, using a 9-month data set from 12 sampling sites in Hong Kong and a 5-month data set from 38 sampling sites in Shenzhen. Our findings will help streamline current sampling practices for SARS-CoV-2 and improve future preparedness for resource allocation in monitoring other pathogens through wastewater surveillance.

## 2 Materials and Methods

### 2.1 Sample Collection

Longitudinal wastewater surveillance on SARS-CoV-2 was conducted in two adjacent major cities, Hong Kong and Shenzhen. The population sizes in 2023 are 7.54 million and 17.66 million for Hong Kong and Shenzhen, respectively (Data source: Google). The sampling period was from February 14, 2022 to November 1, 2022 (9 months) in Hong Kong and is from December 3, 2022 to April 17, 2024 (17 months) in Shenzhen. In Hong Kong, the same sampling campaign was adopted in 12 wastewater treatment plants (WWTPs), i.e., collecting daily 24-h composite samples from the influent of each WWTP. In Shenzhen, wastewater samples were collected from the influent of 38 WWTPs located across 10 different districts. Each district adopted the same sampling practices with a slight difference among different districts. More details were summarized in Table S1, including sampling frequency, sampling types, sampling starting time point, and sampling ending time point. In evaluating Shenzhen’s sampling strategy, we only used a 5-month data set collected from March 21, 2023 to August 28, 2023, because daily sampling was consistently conducted at all 38 sampling points during this period (Table S1).

We have reported the SARS-CoV-2 virus concentration in wastewater collected from 6 WWTPs (FT01, FT02, FT03, NS01, NS02, and NS03 in Shenzhen) from December 3, 2022 to January 31, 2023 (2 months) in a previous publication, which aimed to reveal the Omicron infection trend.^13^ Additionally, another previous study has reported the SARS-CoV-2 virus concentration in wastewater in one WWTP (YT01 in Shenzhen) from February 3, 2023 to January 21, 2024, which aimed to estimate the infection number in the community.^14^ In Hong Kong, our previous study aimed to track the pandemic trend of SARS-CoV-2 and estimate epidemiology indicators (such as prevalence rate and effective reproductive number) by using the same data set in the current study.^12^ Different from the above three published studies, this study aimed to evaluate the sampling strategy in wastewater surveillance by using more comprehensive and long-term longitudinal data sets.

### 2.2 Sample Testing Methods

All wastewater samples underwent virus enrichment, RNA extraction, and RT-qPCR detection according to our previously published studies.^12,13,15^ The polyethylene glycol (PEG) precipitation method was adopted for virus enrichment to obtain concentrated portions from wastewater samples. Subsequently, the concentrated portions were subjected to RNA extraction on the automatic nucleic acid extraction platform (Qiagen QIAcube Connect in Hong Kong, Hybribio HBNP-9601 A in Shenzhen), with a final elution volume of 50 μL. The primer-probe set targeting the SARS-CoV-2 N gene was used for RT-qPCR amplification to detect and quantify the virus signals in wastewater samples. Different versions of PCR instruments were used by the two laboratories, i.e., QuantStudio 7 Flex Real-Time PCR System (Thermo Fisher, USA) in Hong Kong and ABI Prism 7500 Real-Time PCR System (Thermo Fisher, USA) in Shenzhen. The complete details of the protocols and QA/QC procedures have been reported in previous studies,^12,13^ following the Environmental Microbiology Minimum Information (EMMI) guidelines.^16^

### 2.3 Calculation of the Citywide Virus Concentration

After obtaining the virus concentration in each wastewater sample, the citywide virus concentration was calculated based on the flow rate-weighted value by using the eq 1:

1 $$C=\frac{\overset{n}{\underset{i=1}{\sum }}C_{i}\times Q_{i}}{\overset{n}{\underset{i=1}{\sum }}Q_{i}}$$

where

*C*: Daily flow rate-weighted SARS-CoV-2 virus concentration in wastewater at the citywide level;

*i*: The code number of each sampled WWTP;

*C_i_*: Daily SARS-CoV-2 virus concentration in wastewater from each WWTP (copies·L^–1^);

*Q_i_*: Daily flow rate of each sampled WWTP (10^3^ L·day^–1^);

*n*: Number of sampled WWTPs.

### 2.4 Strategies to Conduct Downsampling

Two strategies were used for the downsampling of the number of sites (Figure 1). The first strategy “Enumerative Method” enumerated all the possibilities of site combinations and then identified the best choice with the highest correlation value between the “estimated trend” by using a subgroup of all sampling sites and the “actual trend” by using all sites in a city. This strategy was applicable to the 12 sampling sites in Hong Kong but was not used for the 38 sampling sites in Shenzhen due to the high demand on computational resources. To ensure the feasibility of the method for analyzing large sampling sites in most laboratories, we developed the second strategy “Iterative Hierarchical Method”, which compared all the combinations of randomly removing specific numbers of sites (e.g., *n* = 6) and then selected the best remaining site combination to build a new data set to restart the downsampling procedure. To evaluate the robustness of the second strategy, we employed different numbers of subsampling sites: (1) *n* = 2 for 10 rounds, i.e., the number of sites was changed from 38, 36, 34, 32··· to 18; (2) *n* = 4 for 5 rounds, i.e., the number of sites was changed from 38, 34, 30, 26, 22 to 18; (3) *n* = 5 for 4 rounds, i.e., the number of sites was changed from 38, 33, 28, 23 to 18.

**Figure 1:**
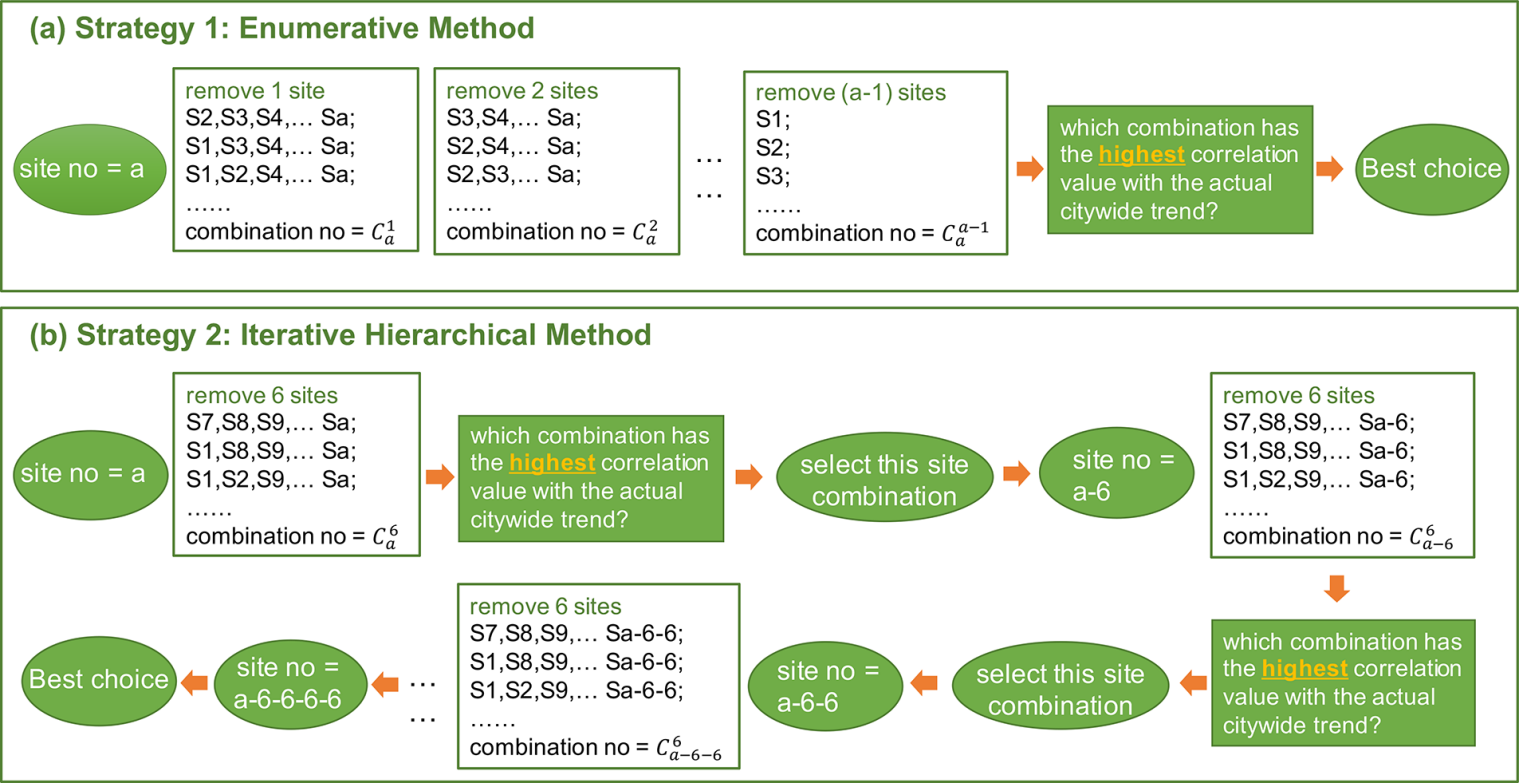
Framework of two downsampling strategies. (a) Strategy 1: Enumerative Method. (b) Strategy 2: Iterative Hierarchical Method, using the removal of 6 sites per batch as an example.

### 2.5 Evaluation Criteria and Statistical Analysis

We aimed to obtain the citywide pandemic trend with fewer sampling sites and lower sampling frequencies. We considered the trend by using all the sampling sites (12 sites in Hong Kong and 38 sites in Shenzhen) as the putative true value, defined as the “actual trend”. The trend using decreased sampling sites was defined as the “estimated trend”. Similarly, in the evaluation of sampling frequencies, the daily sampling results were defined as the “actual trend” and the results from lower sampling frequencies were considered as “estimated trend”. The Pearson correlation coefficient (*r*) between the estimated trend and the actual trend was used as the evaluation criterion for the selection of a subgroup of all the sampling sites for a streamlined sampling strategy, with higher correlation indicating better representativeness. Besides, the paired *t*-test was used to examine if there was a significant difference across different sampling schedules. When the Pearson correlation coefficient is higher than 0.80, and the *p*-value in the paired *t*-test is more than 0.05, we consider it acceptable to use the streamlined sampling practice to reflect the citywide trend. All of the statistical analyses were conducted using MATLAB R2024a.

## 3 Results

### 3.1 Tracking Citywide Trends by Two Downsampling Strategies

We first evaluated the effect of decreasing the number of sampling sites on the correlation between the estimated trend and the actual trend. In Hong Kong, longitudinal monitoring of SARS-CoV-2 concentration was conducted for a period of 9 months at 12 sampling sites (Figure S1a). Based on the Enumerative Method, the removal of 1 to 11 sites from 12 sampling sites led to combination choices ranging from 12 to 924, and the correlation coefficient ranged from 0.3919 to 0.9999 (Figure S2). Using a correlation coefficient of 0.80 as a cutoff, the minimum number of sampling sites was suggested to be 6 from these observations, implying that it was feasible to track a similar trend by halving the number of sampling sites. We selected the combinations of 6 sites with the highest correlation coefficient as the optimized results and found that the optimized 6 sites almost equivalently captured the pandemic trend obtained by the original 12 sites, with a correlation coefficient of 0.9995 (*r* = 0.9995) and showing no significant difference by paired *t*-test (*p* > 0.05) (Figure 2a and b). Among the 924 combinations of selecting 6 sites in Hong Kong, 99.57% (920/924) of the combinations achieved a high correlation coefficient (*r* > 0.8) with the trend by sampling all 12 sites, and most of them (77.16% of 924 combinations) showed no significant difference by paired *t*-test (*p* > 0.05) in the obtained trends. The “worst-case scenario” of the combination of 6 sites could achieve a moderate correlation coefficient of 0.78 and a *p*-value < 0.05 in paired *t*-test, suggesting that the overall upward and downward trend was captured but with a significant difference in the mean value of virus concentration in wastewater samples. These results demonstrated that the application of the Enumerative Method can lead to an optimal solution for a “best choice” to select a subset of the overall sampling sites for capturing the pandemic trends at a reduced cost. We also applied the Iterative Hierarchical Method to Hong Kong’s data sets by using different numbers of subsampling sites (2 or 3 sites) and found that the same 6 sites were selected as the “best choice” of subset sampling sites in the optimized sampling strategies, which highlighted the cross-validation of two downsampling strategies.

**Figure 2:**
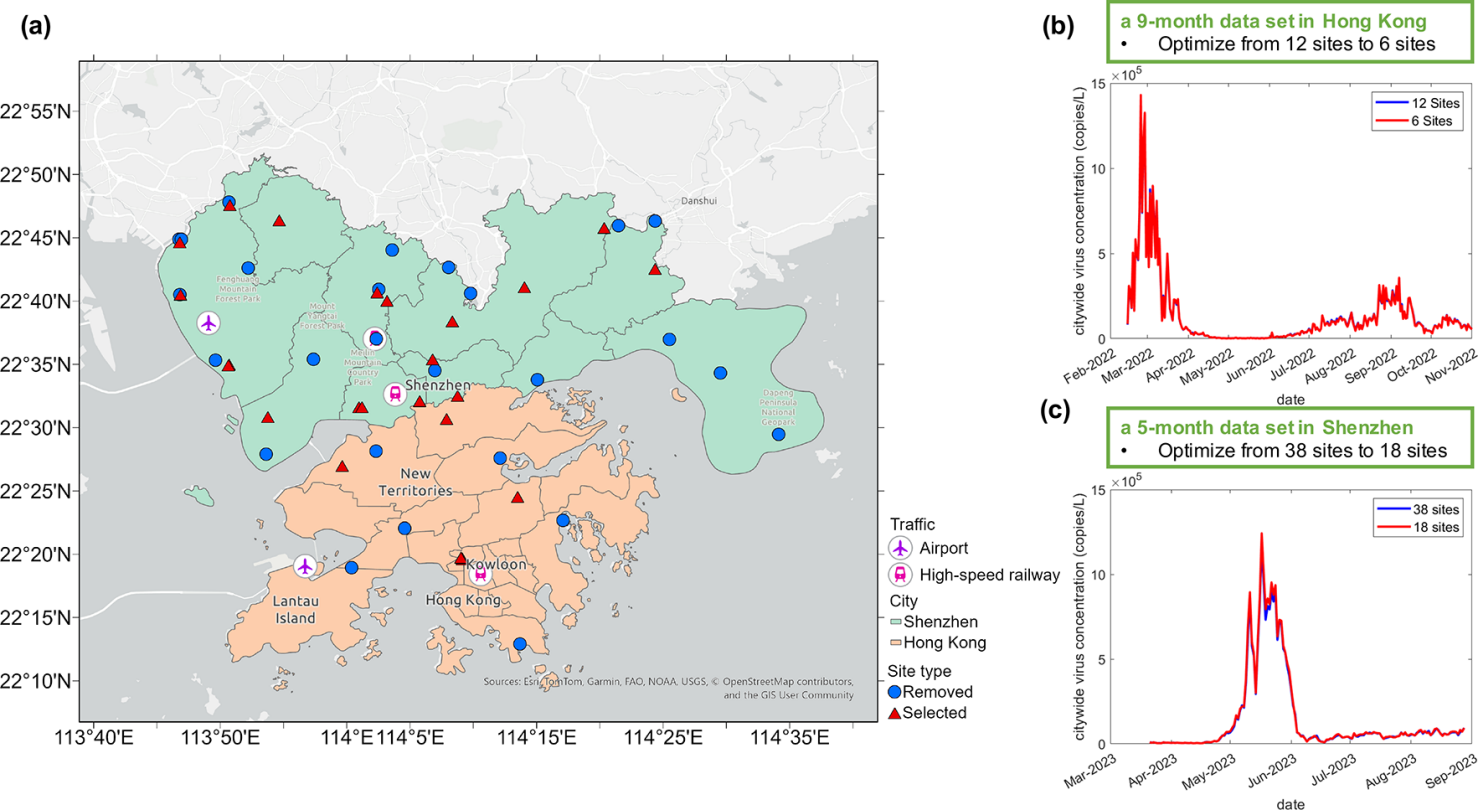
Optimized sampling results in Hong Kong and Shenzhen. (a) The geographical locations of the selected sites and the removed sites. (b) Comparison of obtained trends using the optimized 6 sites and the original 12 sites in Hong Kong. (c) Comparison of obtained trends using the optimized 18 sites and the original 38 sites in Shenzhen.

In Shenzhen, we used a 5-month spatiotemporal SARS-CoV-2 data set from 38 sites for evaluation (Figure S1b). Using the Enumerative Method, the removal of 1 to 5 sites from the 38 sites resulted in combination choices ranging from 38 to 501942, with a correlation coefficient of more than 0.92 (Figure S3). However, with the increase in the number of removed sites, difficulties arose in the calculation procedure due to storage limits in the software. Instead, we employed an Iterative Hierarchical Method to eliminate sampling sites, as illustrated in Figure 1. The results showed that the same 18 sites were selected to reflect the citywide trends, although the number of subsampling sites per batch varied (2, 4, or 5 sites). We observed almost the same trend and a high correlation coefficient between the subset of these 18 sites and the overall 38 sites (*r* = 0.9999 in Pearson correlation, *p* > 0.05 in paired *t*-test, Figure 2a and c), indicating that the optimized site combination could serve as an accurate indicator of the citywide trend at a lower cost. We also compared the results by selecting 18 sites with the highest flow rate and selecting 18 sites randomly to determine if we could use other methods to select the acceptable 18 sites. The comparison results revealed that these selections exhibited a similar trend to the overall 38 sites but displayed notable biases in the calculated virus concentration compared to that obtained using the overall 38 sites, particularly the peak virus concentration (Figure S4). These observations suggested that the Iterative Hierarchical Method was a more recommended strategy for the downsampling of a large number of sampling sites because it can maximize the accuracy and consistency of the obtained trend by using the selected subset of the overall sampling sites.

### 3.2 Factors Influencing Site Selection and Trend Representativeness

To explore the drivers behind the optimization in sampling strategies, we assessed the factors potentially affecting site selection and trend representativeness. As shown in Figure 3, the sampling sites were sorted in descending order based on the median value of daily flow rate during the sampling period. The analysis results revealed a consistent selection pattern in two cities; for example, the selected sites exhibited higher median daily flow rates, along with lower temporal variability compared to removed sites. In contrast, removed sites were associated with lower median flow rates and greater fluctuations over time, which contributed less to the total sampled flow rate in the monitoring network and made them less representative in tracking citywide trends. These findings implied that the flow rate of each sampling site was one of the considered factors in monitoring citywide disease dynamics.

**Figure 3:**
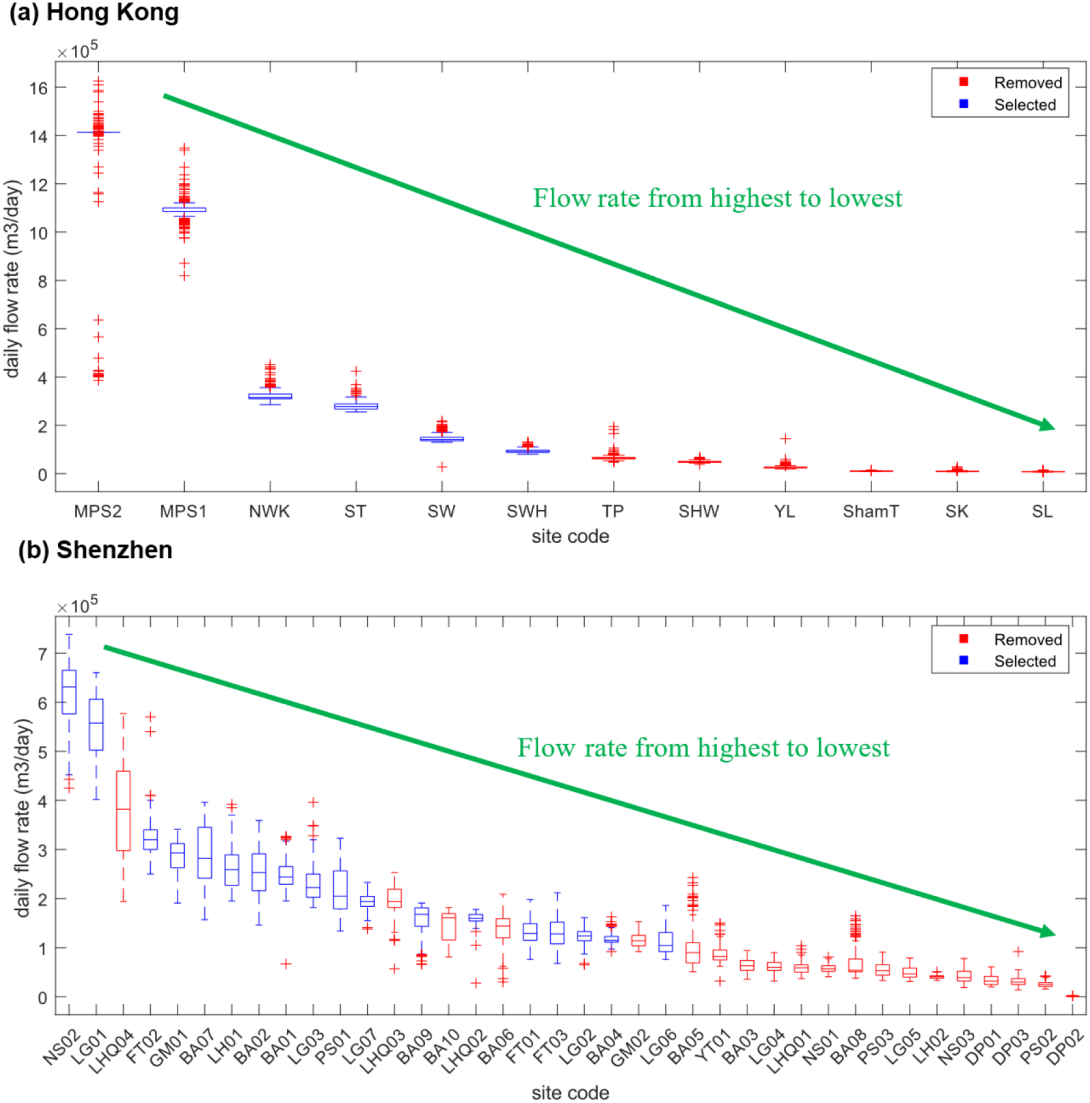
The boxplot of flow rates for selected sites and removed sites in Hong Kong (a) and Shenzhen (b). The sampling sites were sorted in descending order based on the median value of daily flow rate during the sampling period.

Next, we estimated the population size served by each WWTP by dividing the median flow rate of each WWTP by the daily per capita water use (370 L/person/day in Hong Kong), and then assessed the relationship among the number of sampling sites, the serving population ratio, and the representativeness of the trend. The total population size served by each combination was counted during the decrease in sites from 1 to 11 in Hong Kong, defined as the ″serving population ratio”. The Pearson correlation coefficient was used to examine the representativeness of a trend by reduced sites compared to the trend observed by sampling all 12 sites. As shown in Figure S5, the Pearson correlation coefficient generally increased as the serving population ratio rose, indicating that covering a larger proportion of the population tended to improve the representativeness of the trend captured by the monitoring network. However, the results clearly demonstrated that the Pearson correlation coefficient was not determined solely by either the number of sampling sites or the serving population ratio alone. First, for a given number of sampling sites, the correlation coefficient varied considerably across different population coverage levels. Second, even with similar serving population ratios, different combinations of site numbers can lead to differing correlation outcomes, suggesting that the spatial distribution and site-specific factors may also play a critical role.

We also evaluated the geographical pattern of the selected sites and removed sites and found that several selected sites showed proximity to transportation hubs, such as the WWTPs near the southern high-speed railway station of Shenzhen and the Hong Kong high-speed railway station (Figure 2a). However, a comprehensive assessment of spatial representativeness was limited by the current lack of additional data layers (e.g., population density, catchment area of each sampling sites), and additional studies are needed to explore the role of spatial configuration more definitively in the future.

Taken together, these observations underscored that capturing a representative citywide trend was influenced by multiple factors, including sampling intensity (e.g., the number of sampling sites), demographic representativeness (e.g., the serving population ratio), and spatial configuration (e.g., the geographic distribution and intrinsic representativeness of the selected sites). Optimizing a sampling strategy necessitates an integrated framework that concurrently addresses multiple dimensions rather than prioritizing one in isolation.

### 3.3 Recommended Frequency: Three Times per Week for Tracking Citywide Trends

We further investigated the required sampling frequency to capture accurate citywide trends in Hong Kong and Shenzhen. With the decrease in the sampling times per week, a lower correlation coefficient was observed, and higher variations were shown across different combinations of sampling days (Figure 4). The trendlines under different sampling frequencies are compared with those of daily sampling from all sites (Figure 5, Figures S6 and S7). We counted the combination with a high correlation coefficient (*r* > 0.8) and the combination with a significant difference by using a paired *t*-test (*p* < 0.05) under varying sampling frequencies in Hong Kong and Shenzhen. As shown in Table S2, with the increase in sampling frequencies, the percentage of highly correlated combinations (*r* > 0.8) increased, and the percentage of combinations showing statistically significant differences (*p* < 0.05 in paired *t*-test) decreased in the two cities. Higher sampling frequencies are associated with stronger correlations and fewer statistically significant differences in obtained trends for both regions, suggesting that more frequent sampling has a higher probability of obtaining a consistent and comparative trend. The “worst-case scenario” in the current evaluation framework is sampling once per week, which was observed to show 57% of the combinations with a high correlation coefficient (*r* > 0.8) and to have 29% to 43% of the combinations with a significant difference in the obtained trend (*p* < 0.05 in paired *t*-test). Compared to sampling once per week, sampling three times per week demonstrated a clear trend toward a higher proportion of high correlations in the sampling combinations, while the proportion in the sampling combinations showing significant differences was reduced by half, suggesting greater consistency and fewer detectable discrepancies with increased frequency. Therefore, to achieve accurate trendline analysis, sampling three times per week is recommended from our evaluation data sets.

**Figure 4:**
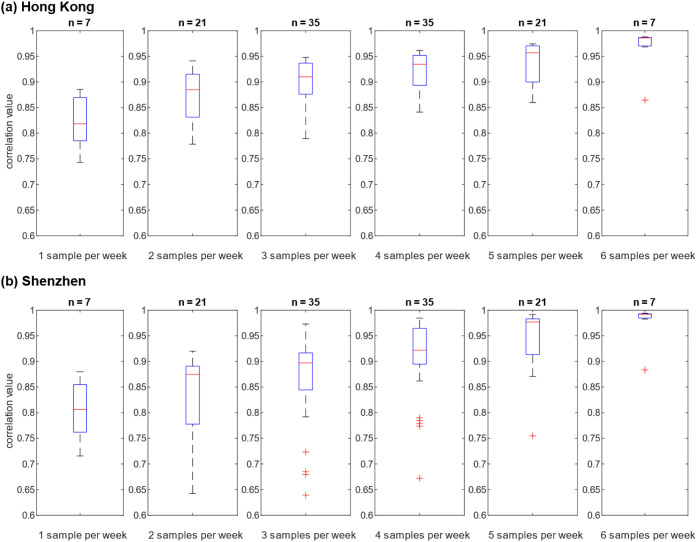
Pearson correlation coefficient of different sampling frequencies in Hong Kong (a) and Shenzhen (b). The number “ $n=C_{7}^{a}$ ” indicates the total combination number at a specific sampling frequency.

**Figure 5:**
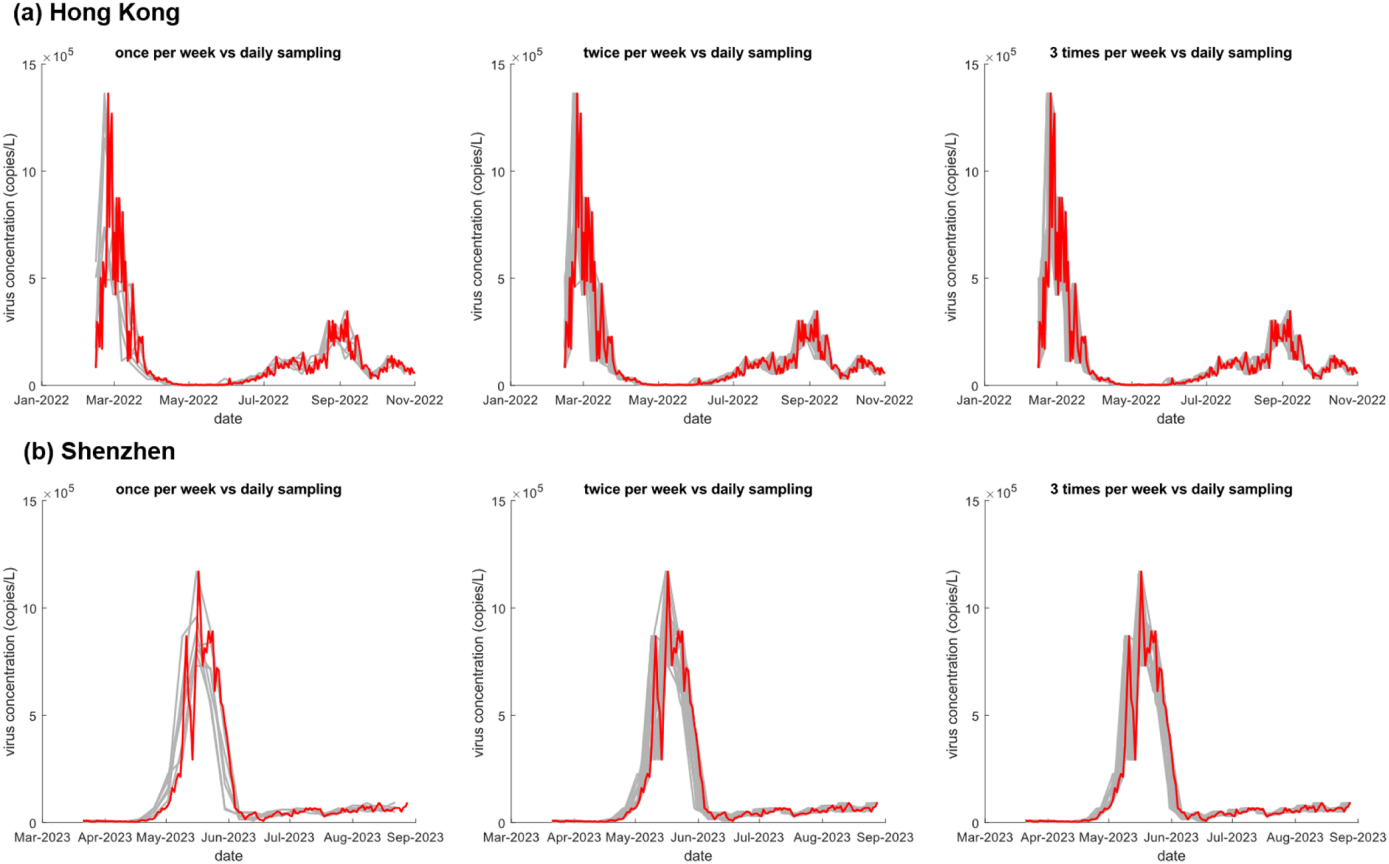
Comparison of citywide trends by different sampling frequencies in Hong Kong (a) and Shenzhen (b). The red line represents the result of daily sampling, while the gray lines represent the results of different combinations of sampling days at a specific sampling frequency (once per week to three times per week). Refer to Figure S5 and Figure S6 for these plots at a higher sampling frequency.

## 4 Discussion

Wastewater surveillance presents significant public health value in reflecting the spatiotemporal development of COVID-19 trends and identifying other potential emerging pathogens.^17–19^ Numerous wastewater surveillance networks have been launched to help public health officials take timely mitigation efforts, such as the National Wastewater Surveillance System (NWSS) program across the United States^20^ and the EU Wastewater Observatory for Public Health program in Europe.^21^ At the same time, running a WBE campaign relies on significant resources (i.e., money, labors, reagents, materials, etc.) and requires continuous funding and support from different stakeholders.^22^ The smart sampling strategy for wastewater surveillance is a trade-off between achieving monitoring purposes and reducing unnecessary sampling practices to maximize the value of WBE and reallocate limited resources more effectively. There are several sampling recommendations to monitor trends from the published WBE guidance, such as sampling weekly by the World Bank^23^ and sampling twice to three times per week by the Centers for Disease Control and Prevention (CDC).^24,25^ However, most reported studies on sampling strategies are generated from empirical observations, lacking robust evidence-based data sets to support their opinions. In this study, we provided insights into achieving cost-effective sampling strategies based on the comprehensive analysis of two long-term longitudinal data sets in Hong Kong and Shenzhen.

First, two downsampling strategies were provided to identify the selected sites and the removed sites in capturing citywide trends. Strategy 1, the Enumerative Method, is straightforward and compares all of the combinations to identify the best choice. It aided in reducing the sampling sites by half while maintaining the same citywide trends in Hong Kong’s data sets. However, technical issues arose during the adoption of the calculation procedure to more sampling sites, such as the 38 sites in Shenzhen. To tackle this problem, Strategy 2, the Iterative Hierarchical Method, was proposed to decrease the number of sampling sites in different batches. This method was feasible and robust in achieving good performance in Shenzhen’s data sets (Figure 2c). To the best of our knowledge, there has been no reported study on the optimization of the WBE sampling strategy yet. The establishment and demonstration of these two downsampling strategies are crucial to providing reference case studies for other regions to streamline their current wastewater surveillance practices for reducing testing costs, especially as the incidence of COVID-19 is gradually changing from pandemic to endemic.

Second, factors influencing site selection and trend representativeness were evaluated during the optimization of sampling strategies, including flow rate, the number of sampling sites, served population size, and geographical locations. The selected sites were characterized by higher median daily flow rates and lower temporal variability, which generally provided stronger and more stable signals, thereby supporting their utility in reflecting citywide wastewater trends. Previous studies have also put forward a similar opinion that sampling sites should be prioritized to be arranged in WWTPs with high flow rates and high population coverage,^26–28^ aiming to increase their capacity to integrate signals from larger populations while minimizing noise from hydraulic fluctuations. However, it should be noted that the population coverage or flow rate at sampling sites should not be expanded without limits but rather reasonably determined based on surveillance purposes, method detection limits, and community prevalence rates. Several researchers proposed a model-based method to evaluate the possibilities of monitoring targeted viruses in wastewater under different scenarios,^29–31^ which may assist in preliminary evaluations before implementing wastewater surveillance practices. In addition, we found neither the number of sites nor the population coverage alone can consistently predict trend representativeness, supporting the idea that multiple factors contribute to trend representativeness. For instance, different spatial combinations of sites yielding similar population coverage produced substantially different correlation outcomes (Figure S5). Therefore, an effective sampling strategy was suggested to balance these factors, such as sampling sufficient sites to ensure statistical robustness, covering adequate population sizes to represent the community, and performing a deliberate spatial layout that captures relevant heterogeneity (e.g., urban cores, transportation hubs, and residential areas).

Third, we evaluated the Pearson correlation coefficient and paired *t*-test under varying sampling frequencies in currently available data sets. In this study, for tracking representative and accurate trendlines, it was recommended to conduct sampling at WWTPs three times per week (Figure 4, Table S2), which was more frequent than our previous evaluation result (twice per week) for the collected data sets from December 2020 to June 2021 in Hong Kong^10^ and another study sampling twice per week from September 2020 to October 2020 in Wisconsin, USA.^11^ This inconsistency may be attributed to the difference in circulating virus variants (from Alpha to Omicron), suggesting that a higher sampling frequency in wastewater surveillance is needed to keep pace with the shorter incubation time and faster transmission pattern of Omicron variants.^32^ Moreover, as shown in Figure 5, different sampling frequencies captured comparable transmission trends but presented significant differences in the specific values of the observed citywide virus concentrations, especially during the peak period. Although this result may not have a significant impact on observing the overall trend of the pandemic, it will considerably influence the assessment of risk levels of virus transmission and the estimation of multiple epidemiological parameters, such as prevalence rate and incidence rate. In addition, the sampling frequency should not be treated as a fixed parameter but must be dynamically adjusted in response to the evolving pandemic situation. The selection of the sampling strategy is a trade-off between the required representativeness of trends, the needed accuracy of measurements, and the availability of resources (e.g., money, labor, logistical arrangements at sampling sites and sampling frequencies). Based on the available data sets in this study, sampling three times per week is recommended for routine nonepidemic monitoring, and the sampling frequency could be increased/decreased with reference to other epidemiological information. To ensure adaptive surveillance, multiple epidemiological indicators from available information, including (but not limited to) herd immunity effects, the emergence of new variants, and shifts in surveillance objectives, should be regularly evaluated. This iterative adjustment process may enhance the timeliness and precision of public health interventions.

There are several limitations in this study. First, owing to the unavailability of data, this study lacks an assessment of other potential contributing factors to site selection and trend representativeness, as demonstrated in previous work on chemical consumption surveillance,^33^ such as population behavior, population density, and the proportion of the floating population. Further research on these aspects will help to understand the effect of sociodemographic or socioeconomic factors on virus transmission patterns and provide guidance for developing a future WBE network. Second, this work is limited in terms of the virus types and the number of cities, serving as a pilot study on the assessment of sampling strategies for SARS-CoV-2 wastewater surveillance in only two cities. It is suggested that the developed framework could be applied to evaluate other viruses (such as influenza virus and diarrhea viruses) in more regions, which would benefit the understanding of sampling strategies for a wider range of pathogens. Despite the unavailability of data sets to evaluate other pathogens, the main contribution of this work is to provide a practical downsampling framework and its proof-of-concept application using data sets from two cities, which could be followed by other cities to streamline their current sampling practices with reduced costs for sustainable, cost-effective longitudinal monitorings beyond the pandemic. Third, it should be emphasized that the sampling strategy always depends on the purposes of wastewater surveillance. This study focused on the “best choice” to achieve the objective of tracking pandemic trends, as this is one of the major roles of wastewater surveillance in complementing public health interventions. However, the “best choice” of sampling strategies may change when the focus shifts to other surveillance purposes, such as giving early warning signals, uncovering hidden cases in communities, revealing new virus variants, and evaluating vaccination effects. For example, several studies have designed wastewater surveillance by sampling at upstream manhole sites to trace previously unsuspected patients,^34^ and sampling on college campuses to reveal the effect of mass vaccination^35^ or to alert school management,^36^ which are beyond the evaluation scope in this study.

To sum up, wastewater surveillance has been proven to be a useful tool to offer a comprehensive view of the infection burden at the population level for effective public health interventions. However, the operating costs remain a problem for long-term monitoring during the postpandemic era. In this study, we proposed two methods for reducing the number of sampling sites and the sampling frequency and applied them to two longitudinal data sets in Hong Kong and Shenzhen. Multiple factors contributing to site selection and trend representativeness were evaluated, including the flow rate of sampling sites, sampling intensity (number of sites), demographic representativeness (population coverage), and geographical locations. In addition, sampling three times per week could effectively track the Omicron outbreak in this study, and sampling frequency requires timely adaptation contingent upon the latest epidemiological trends from other available surveillance tools. The observations from this study help to guide practices in wastewater surveillance designs and enable researchers to optimize sampling strategies for their own specific surveillance purposes. Such retrospective studies will be beneficial in building a global, informative, and cost-effective wastewater surveillance system to implement evidence-based interventions for controlling pathogen outbreaks in the future.

## Supplementary Material



## Data Availability

All data related to the analysis of the results and discussion in the article are available in the main text and the Supporting Information. All the raw data are available from the corresponding author upon reasonable request.

## Notes

Additional details on methods and results, including SARS-CoV-2 virus concentrations from each WWTP (Figure S1), Pearson correlation coefficients for different removed site numbers or different population sizes (Figures S2, S3, S5), obtained trends from different site combinations or different sampling frequencies (Figures S4, S6, S7), sampling information (Table S1), and Pearson correlation coefficients and paired *t-*test results for different sampling frequencies (Table S2) (PDF)
