# Supplementary material for: Cost-Effective Sampling Strategies for Wastewater Surveillance: A Large-Scale Longitudinal Study in Hong Kong and Shenzhen
Source: Environ Sci Technol. 2026 Mar 6;60(11):8315–25. doi: 10.1021/acs.est.5c02652 (PMC13019662; doi:10.1021/acs.est.5c02652)
Supplement: Supplementary file 1 [file es5c02652_si_001.pdf]

Supporting information

for

Cost-Effective Sampling Strategies for Wastewater Surveillance: A Large-Scale Longitudinal Study in Hong Kong and Shenzhen

Xiawan Zheng[1#], Yinghui Li[2, 8#], Yu Deng[1], Bincai Wei[6], Xiaoqing Xu[1], Chen Du[2], Guixian Luo[2], Miaomiao Luo[2], Xiuyuan Shi[6], Yuejing Peng[2], Shuxian Li[1], Jiahui Ding[1], Bingjie Xue[1, 3], Yanping Mao[3], Qinghua Hu[2, 6]*, and Tong Zhang[1, 4, 5, 7]*

Affiliations:

[1] Environmental Microbiome Engineering and Biotechnology Laboratory, Center for Environmental Engineering Research, Department of Civil Engineering, The University of Hong Kong, Pokfulam, Hong Kong SAR 999077, China.

[2] Shenzhen Center for Disease Control and Prevention, Shenzhen 518073, China.

[3] College of Chemistry and Environmental Engineering, Shenzhen University, Shenzhen 518071, China

[4] School of Public Health, LKS Faculty of Medicine, The University of Hong Kong, Pokfulam, Hong Kong SAR 999077, China

[5] Department of Environmental Science and Engineering, Macau University of Science and Technology, Taipa, Macau SAR 999078, China

[6] School of Public Health and Emergency Management, Southern University of Science and Technology, Shenzhen 518055, China

[7] Shenzhen Innovation and Research Institute, The University of Hong Kong, Shenzhen 518057, China

[8] School of Public Health, Southern Medical University, Guangzhou 510515, China

#These two authors contributed equally to this work.

*Corresponding author. E-mail: zhangt@hku.hk (Tong Zhang); huqinghua03@163.com (Qinghua Hu)

Contents

The file contains seven Figures and two Tables in a total of 13 pages.

Contents

List of Figures

Figure S1 SARS-CoV-2 virus concentration in wastewater from each WWTP.

Figure S2 Pearson correlation coefficient of different removed site numbers in Hong Kong.

Figure S3 Pearson correlation coefficient of different removed site numbers in Shenzhen.

Figure S4 Comparison of citywide trends by different sites combinations in Shenzhen.

Figure S5 Evaluation on the influence of population sizes on the Pearson correlation coefficient by varying combinations in Hong Kong.

Figure S6 Comparison of citywide trends by different sampling frequencies in Hong Kong.

Figure S7 Comparison of citywide trends by different sampling frequencies Shenzhen.

List of Tables

Table S1 Sampling information of wastewater surveillance in Shenzhen.

Table S2 Comparison of the Pearson correlation coefficient (*r*) and paired *t*-test in different sampling frequencies.

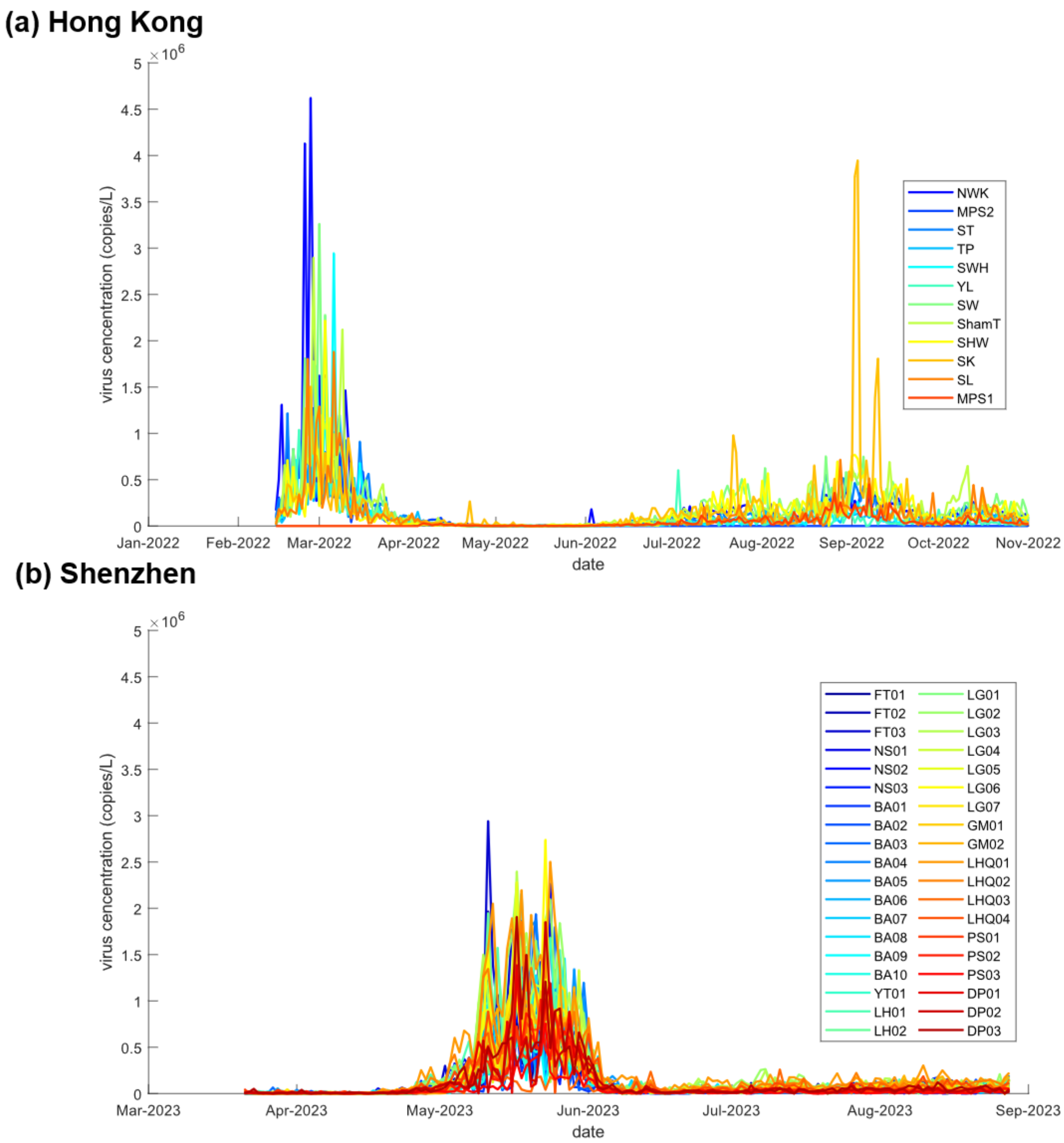

Figure S1 SARS-CoV-2 virus concentration in wastewater from each WWTP. **(a)** 12 sites in Hong Kong. **(b)** 38 sites in Shenzhen.

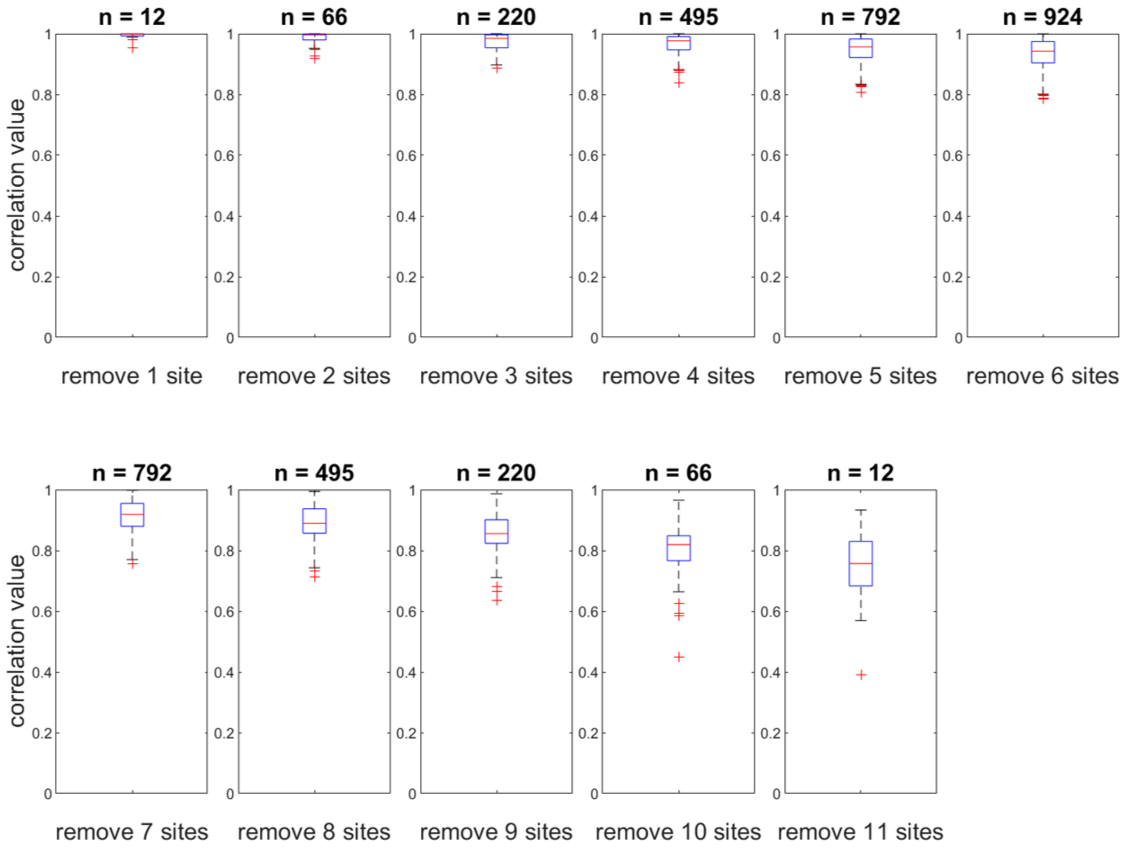

Figure S2 Pearson correlation coefficient of different removed site numbers in Hong Kong. There are 12 sampling sites in Hong Kong. The number "n = C_{12}^{N}" (N is from 1 to 11) indicated the total combination number at a specific removed site number.

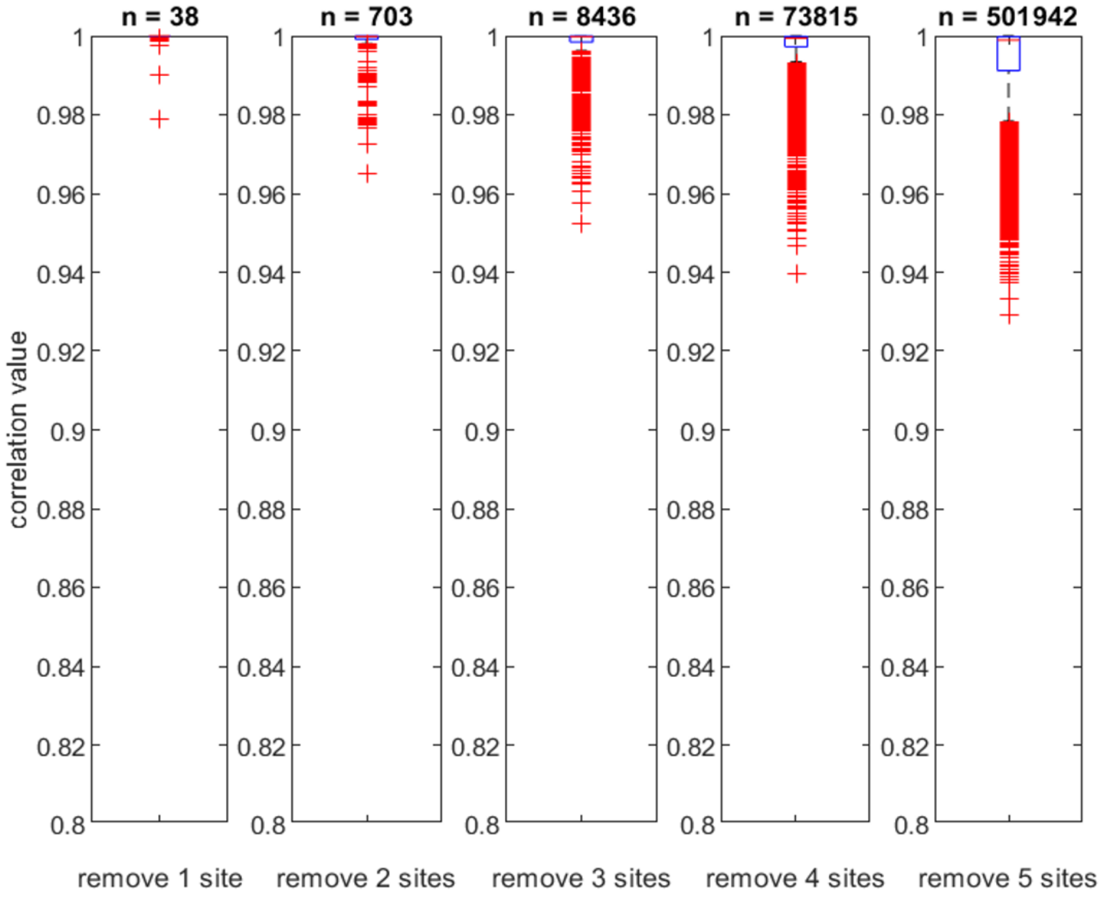

Figure S3 Pearson correlation coefficient of different removed site numbers in Shenzhen. There are 38 sampling sites in Shenzhen. The number “n = C_{38}^{N}” (N is from 1 to 5) indicated the total combination number at a specific removed site number.

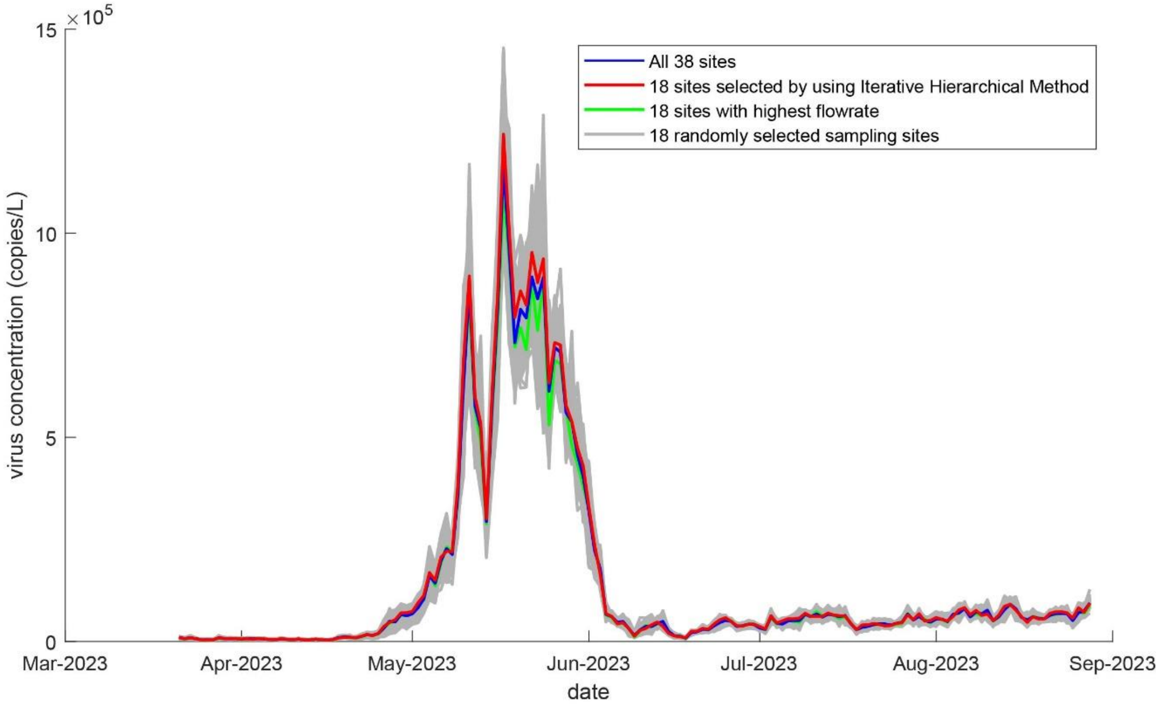

Figure S4 Comparison of citywide trends by different sites combinations in Shenzhen.

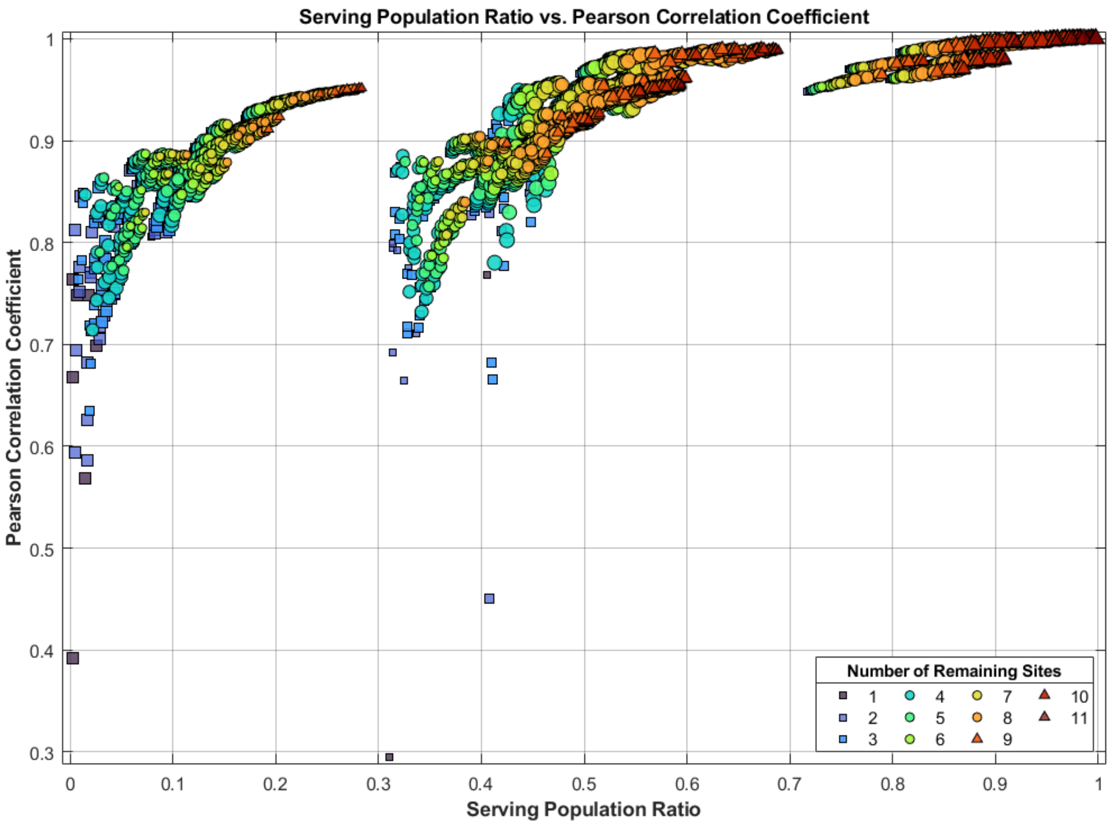

Figure S5 Evaluation on the influence of population sizes on the Pearson correlation coefficient by varying combinations in Hong Kong.

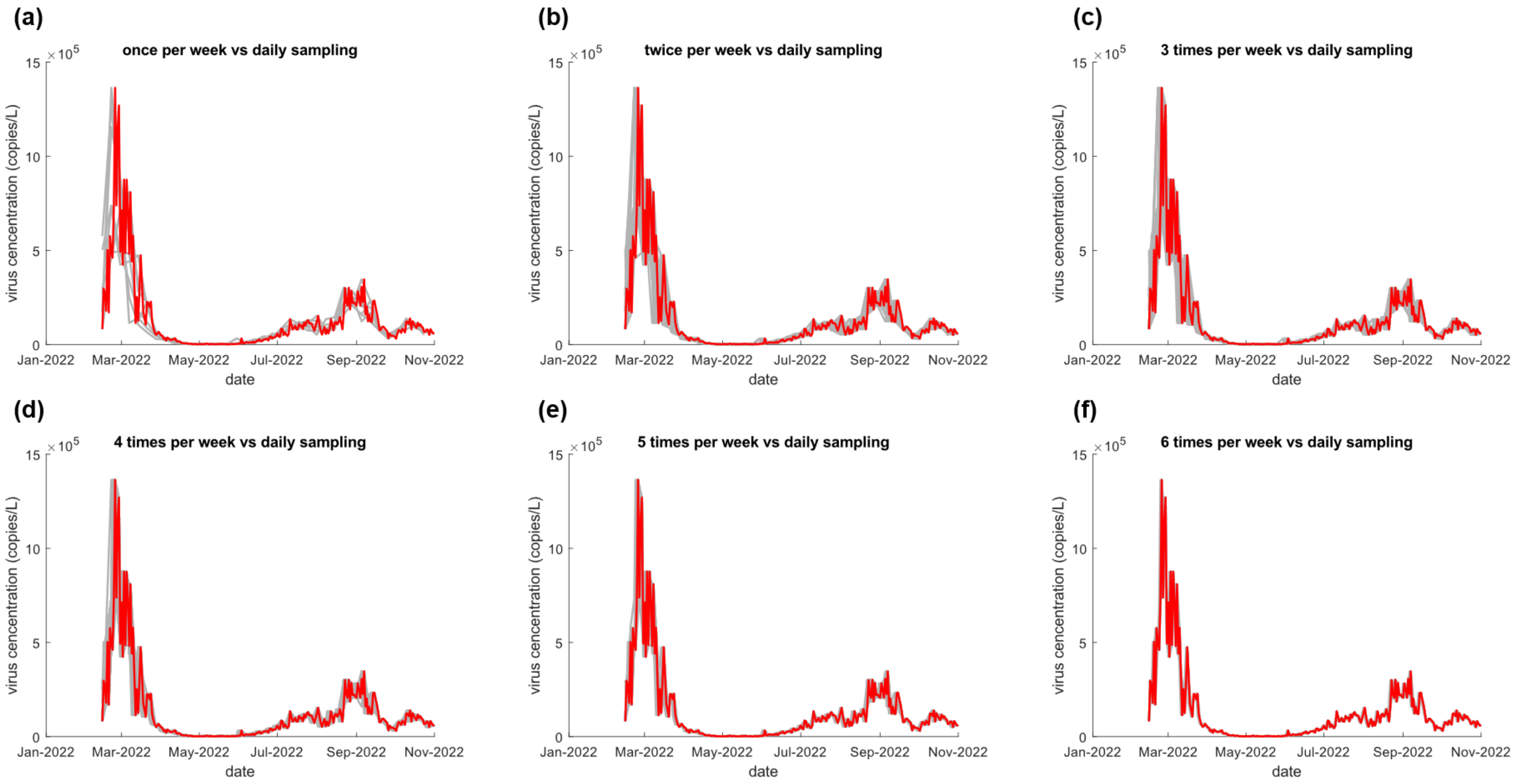

Figure S6 Comparison of citywide trends by different sampling frequencies in Hong Kong. The red line represents the result by daily sampling, while the gray lines represent the results by different combinations of sampling days at a specific sampling frequency (from once per week to 6 times per week).

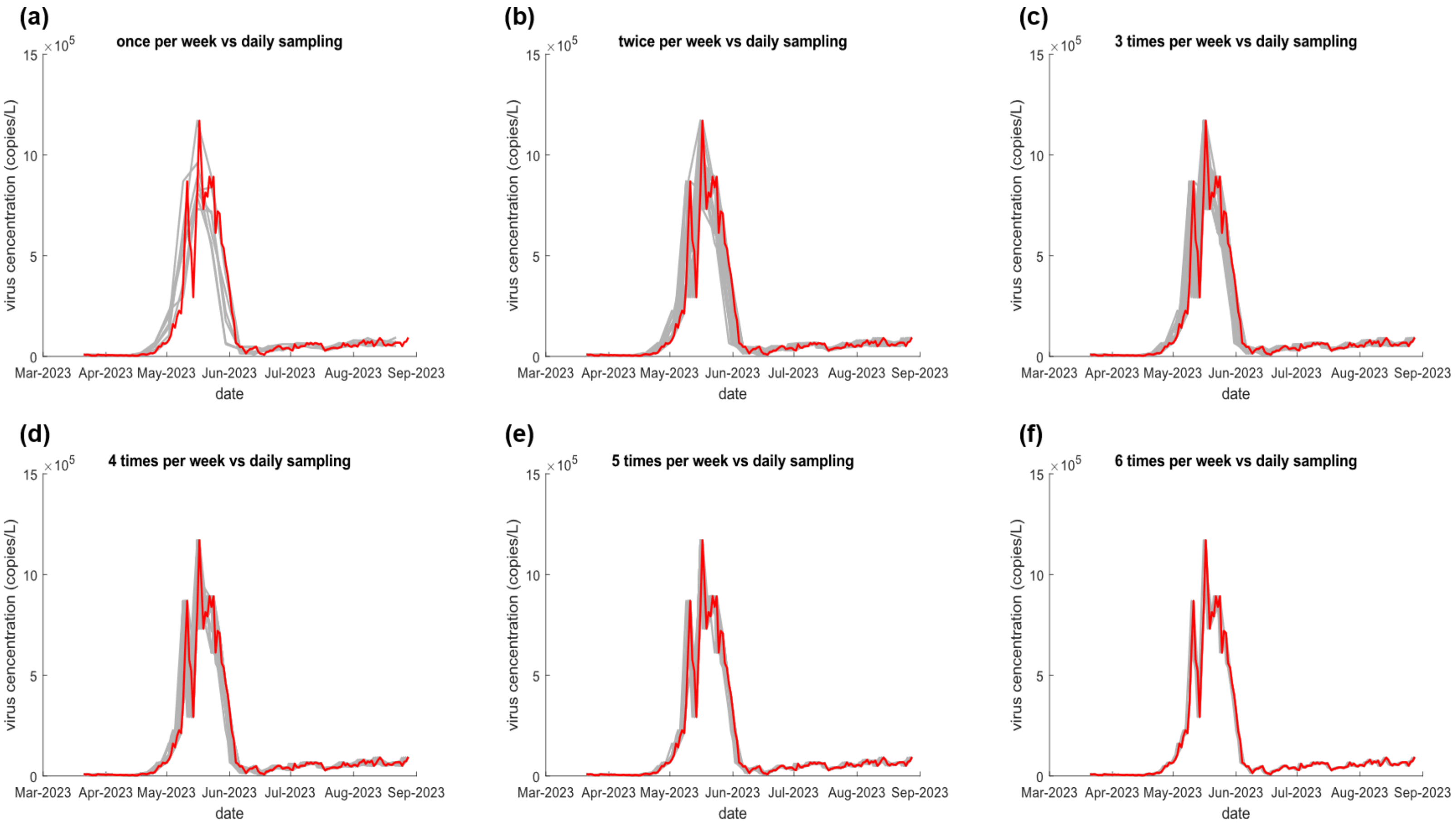

Figure S7 Comparison of citywide trends by different sampling frequencies in Shenzhen. The red line represents the result by daily sampling, while the gray lines represent the results by different combinations of sampling days at a specific sampling frequency (from once per week to 6 times per week).

Table S1 Sampling information of wastewater surveillance in Shenzhen.

District	Total site no.	Sites code	Sampling period	Sampling type	Sampling starting timepoint	Sampling ending timepoint	Sampling frequency
Futian	3	FT01, FT02, FT03	2022/12/3 - 2022/12/27	3 h composite samples	7:00	10:00	daily
			2022/12/28 - 2023/8/28	24 h composite samples	7:00	7:00	daily
			2023/8/30 - 2023/12/31	24 h composite samples	7:00	7:00	bidaily
			2024/1/1 - 2024/4/17	24 h composite samples	0:00	0:00	twice per week
Nanshan	3	NS01, NS02, NS03	2022/12/3 - 2023/1/20	3 h composite samples	7:00	10:00	daily
			2023/1/21 - 2023/8/28	24 h composite samples	10:00	10:00	daily
			2023/8/30 - 2023/12/31	24 h composite samples	10:00	10:00	bidaily
			2024/1/1 - 2024/4/17	24 h composite samples	0:00	0:00	twice per week
Baoan	10	BA01, BA02, BA03, BA04, BA05, BA06, BA07, BA08, BA09, BA10	2022/12/22 - 2023/1/2	3 h composite samples	7:00	10:00	daily
			2023/1/3 - 2023/8/28	24 h composite samples	10:00	10:00	daily
			2023/8/30 - 2023/12/31	24 h composite samples	10:00	10:00	bidaily
			2024/1/1 - 2024/4/17	24 h composite samples	0:00	0:00	twice per week
Yantian	1	YT01	2022/12/26 - 2023/8/28	24 h composite samples	10:00	10:00	daily
			2023/8/30 - 2023/12/31	24 h composite samples	10:00	10:00	bidaily
			2024/1/1 - 2024/4/17	24 h composite samples	0:00	0:00	twice per week
Luohu	2	LH01, LH02	2023/1/1- 2023/8/28	24 h composite samples	9:00	9:00	daily
			2023/8/30 - 2023/12/31	24 h composite samples	10:00	10:00	bidaily
			2024/1/1 - 2024/4/17	24 h composite samples	0:00	0:00	twice per week
Longgang	7	LG01, LG02, LG03, LG04, LG05, LG06, LG07	2023/1/1 - 2023/8/28	24 h composite samples	0:00	0:00	daily
			2023/8/30 - 2023/12/31	24 h composite samples	0:00	0:00	bidaily
			2024/1/1 - 2024/4/17	24 h composite samples	0:00	0:00	twice per week

District	Total site no.	Sites code	Sampling period	Sampling type	Sampling starting timepoint	Sampling ending timepoint	Sampling frequency
Guangming	2	GM01, GM02	2023/1/1 - 2023/8/28	24 h composite samples	0:00	0:00	daily
			2023/8/30 - 2023/12/31	24 h composite samples	0:00	0:00	bidaily
			2024/1/1 - 2024/4/17	24 h composite samples	0:00	0:00	twice per week
Longhua	4	LHQ01, LHQ02, LHQ03, LHQ04	2023/1/4 - 2023/8/28	24 h composite samples	10:00	10:00	daily
			2023/8/30 - 2023/12/31	24 h composite samples	10:00	10:00	bidaily
			2024/1/1 - 2024/4/17	24 h composite samples	0:00	0:00	twice per week
Pingshan	3	PS01, PS02, PS03	2023/1/1 - 2023/8/28	24 h composite samples	10:00	10:00	daily
			2023/8/30 - 2023/12/31	24 h composite samples	10:00	10:00	bidaily
			2024/1/1 - 2024/4/17	24 h composite samples	0:00	0:00	twice per week
Dapeng	3	DP01, DP02, DP03	2023/1/1 - 2023/8/28	24 h composite samples	10:00	10:00	daily
			2023/8/30 - 2023/12/31	24 h composite samples	10:00	10:00	bidaily
			2024/1/1 - 2024/4/17	24 h composite samples	0:00	0:00	twice per week

Table S2 Comparison of the Pearson correlation coefficient (*r*) and paired *t*-test in different sampling frequencies.

Sampling frequency	Total number of combinations	Number of combinations with a $r > 0.8$ in Pearson correlation	Percentage of high correlation	Number of combinations with a $p < 0.05$ in paired *t*-test	Percentage of significant differences
**Hong Kong**					
1 sample per week	7	4	57%	2	29%
2 samples per week	21	19	90%	4	19%
3 samples per week	35	34	97%	3	9%
4 samples per week	35	35	100%	1	3%
5 samples per week	21	21	100%	0	0%
6 samples per week	7	7	100%	0	0%
**Shenzhen**					
1 sample per week	7	4	57%	3	43%
2 samples per week	21	15	71%	5	24%
3 samples per week	35	30	86%	6	17%
4 samples per week	35	30	86%	5	14%
5 samples per week	21	20	95%	2	10%
6 samples per week	7	7	100%	0	0%
